# *Plantago lagopus* extract as a green fungicide induces systemic resistance against *Rhizoctonia* root rot disease in tomato plants

**DOI:** 10.3389/fpls.2022.966929

**Published:** 2022-08-08

**Authors:** Said I. Behiry, Abdulaziz A. Al-Askar, Seham A. Soliman, Fatimah O. Alotibi, Adriana Basile, Ahmed Abdelkhalek, Mohsen Mohamed Elsharkawy, Mohamed Z. M. Salem, Elsayed E. Hafez, Ahmed A. Heflish

**Affiliations:** ^1^Department of Agricultural Botany, Faculty of Agriculture (Saba Basha), Alexandria University, Alexandria, Egypt; ^2^Department of Botany and Microbiology, College of Science, King Saud University, Riyadh, Saudi Arabia; ^3^Department of Plant Protection and Biomolecular Diagnosis, ALCRI, City of Scientific Research and Technological Applications, Alexandria, Egypt; ^4^Department of Botany and Microbiology, College of Science, King Saud University, Riyadh, Saudi Arabia; ^5^Department of Biology, University of Naples Federico II, Naples, Italy; ^6^Department of Agricultural Botany, Faculty of Agriculture, Kafrelsheikh University, Kafr El-Sheikh, Egypt; ^7^Department of Forestry and Wood Technology, Faculty of Agriculture (El-Shatby), Alexandria University, Alexandria, Egypt

**Keywords:** *Plantago lagopus*, *Rhizoctonia solani*, tomato, defense-related genes, gene expression, HPLC

## Abstract

Extensive use of chemical control agents and fungicides typically leads to numerous risks to human health and the environment. Using plant extracts as natural substances represents a dual key for the environment and sustainable food production, as it reduces the input of synthetic pesticides into the environment and/or controls plant pathogens. For the first time, a *Plantago lagopus* ethanolic extract has been characterized and evaluated for its protective and curative effects against *Rhizoctonia solani* in tomato plants. The results showed that *P. lagopus* extract (10 μg/ml) completely inhibited *R. solani* mycelial growth *in vitro*. At 20 days of post fungal inoculation, the results demonstrated that using *P. lagopus* extract (100 μg/ml) *in vivo* enhanced tomato plant growth by significantly increasing shoot and root parameters in protective and curative treatments. Furthermore, the protective and curative treatments significantly reduced the disease index by 18.66 and 38.66%, respectively. Induction of systemic resistance with upregulation of *PR-1* and *PR-2* and a significant increase in the transcriptional levels of *PR-3* and *CHS* in all *P. lagopus* extract-treated tomato plants were reported compared to untreated plants. HPLC analysis showed that the most common polyphenolic components detected in *P. lagopus* extract were rutin (74206.3 mg/kg), naringenin (2388.74 mg/kg), quercetin (1249.13 mg/kg), and *p*-hydroxybenzoic acid (1035.87 mg/kg). In addition, the ellagic acid (798.47 mg/kg), vanillic acid (752.55 mg/kg), catechol (648.89 mg/kg), cinnamic acid (332.51 mg/kg), ferulic acid (296.32 mg/kg), benzoic acid (295.95 mg/kg), and chlorogenic acid (116.63 mg/kg) were also reported. Our study is the first to show that *P. lagopus* extract can help plants fight off *R. solani* fungal infection. Furthermore, the findings imply that using the *P. lagopus* extract as a natural biocontrol agent could be a sustainable strategy to manage plant fungal diseases.

## Introduction

Tomatoes (*Lycopersicon esculentum* L.) are among the most remunerative and widely cultivated vegetables worldwide. It is in the family Solanaceae and has tryptophan and tomatin, which are very good for human health. Tomatin is a main component of the glycoalkaloid. Tomatoes are the first-ranked processed vegetable and rank second only to potatoes in the world growing area. According to FAOSTAT (2017), global tomato production is about 161.7 million metric tons with a value of $59 billion. Harmful microorganisms threaten crop production and environmental balance (Sabuquillo et al., 2006). Various pathogens (*Rhizoctonia*, *Verticillium*, *Fusarium,* and *Pythium*) are responsible for the soil-borne fungal diseases, damping-off, and wilt of tomato seedlings (Lucas et al., 1992; Kuprashvili, 1996). *Rhizoctonia solani* is a common soil-borne fungal pathogen that causes seedling damping-off and root rot in tomatoes (Gondal et al., 2019). Damping-off caused by *Rhizoctonia solani* is one of the most devastating tomato diseases managed by fungicides. Different symptoms are associated with damping-off, which causes the death of some seedlings in the population (Channa et al., 1995). Symptoms start with round spots on seedlings, then stem abrasions at the surface level of seedlings (Channa et al., 1995; Heflish et al., 2021). Because physiological pathogen races have become more resistant to fungicides and factors like fungicide residues and human health issues, it is hard to develop new ways to manage plant diseases.

The concept of biocontrol has sparked a significant technological, economic, and political discussion that seeks to promote environmentally sustainable agriculture at a lower cost to the environment. Accordingly, some nations have created a protection plan that minimizes pesticide use by approximately 50%. From this perspective, it appears vital to increase our understanding of biocontrol to improve its application and effectiveness. For all these reasons, research is making good progress toward a biological control point of view that could be combined with other methods to make a good plan for fighting plant diseases (Abo-Zaid et al., 2021). Many biocontrol agents are reported to fight *R. solani* pathogens such as Bacillus subtilis and *Trichoderma* species, which stop the pathogen’s growth or progress with different strategies like production of siderophores, cell lysis enzymes, or by direct intact (hyperparasitism; Wu et al., 2019; El-Benawy et al., 2020; Heflish et al., 2021). Also, various plant extracts are reported for their antifungal activity against *R. solani,* including a diverse array of bioactive chemicals that are well-known for their antimicrobial and antifungal activity without causing phytotoxic effects (Al-Askar and Rashad, 2010; Santas et al., 2010; Abd-El-Khair and El-Gamal Nadia, 2011; Hamza et al., 2016; Koka et al., 2017; Parveen et al., 2017). The main reasons for using plant extracts or essential oils as antifungal agents are that they come from nature, and pathogens are less likely to become resistant to them. Since plant products are easy to turn into common organic materials, they may have less of an effect on the physiological processes of plants and less of an impact on the environment than their synthetic alternatives (eco-friend; Nazzaro et al., 2017; Abdelkhalek et al., 2021).

Many plants and their extracts have been evaluated for their antimycotic activities. They are known to have good antifungal activities against plant pathogenic fungi (Shuping and Eloff, 2017), such as *Plantago* plants which have been used as anti-inflammatory and asthmatic medications in Asia and Europe (Nishibe et al., 1995). *Plantago lanceolata* L. extract was used as an antifungal against fungi such as *Alternaria alternata*, *Mucor piriformis,* and *Penicillium expansum* (Parveen et al., 2014). Also, *Plantago* extract was used as an inhibitor for spore germination of the *Colletotrichum gloeosporioides* Penz, which is the main causal agent of blister spot disease in coffee trees (Silva et al., 2008). Moreover, (Klironomos, 2003) postulated that *P. lanceolata* protects the treated plants against fungal infection. Four iridoid glucosides, i.e., plantamajoside, luteolin-7-O-monoglucoside, chlorogenic acid, and rosmarinic acid, were isolated from the aerial parts of *P. lagopus* (Velázquez-Fiz et al., 2000). Moreover, *p*-hydroxybenzoic, chlorogenic, gallic, and vanillic acids or apigenin, luteolin, and luteolin-7-O-glucoside were common compounds between *P. altissima* and *P. lanceolata* extracts (Beara et al., 2012).

In addition, the plant extracts contain various chemicals, including plant hormones, minerals, antioxidants, and osmoprotectants, which effectively boost the defense mechanisms (antioxidant enzymes) of plants against environmental challenges and play a crucial role in promoting plant growth (Desoky et al., 2019). These extracts possess protective enzymes and activate pathogenesis-related proteins, thereby inhibiting the progression of the disease. It was reported that these extracts might stop the spread of disease by activating the body’s defenses and causing systemic resistance (Prasannath, 2017). Recognizing the importance of screening and identifying new plant extracts with strong antifungal activities for agricultural applications, we hypothesized that *P. lagopus* extract can fight off the *R. solani* pathogen *in vitro*, improve tomato growth parameters *in vivo*, and could induce resistance against *Rhizoctonia* root rot disease. To test this hypothesis, we evaluated the growth inhibitory activity of *P. lagopus* ethanolic extract against *R. solani*, its effects on growth parameters, total chlorophyll, phenolic content, and defense-related gene expression levels, including *PR-1*, *PR-2*, *PR-3*, and *CHS* in tomato plants. Also, use HPLC to figure out the *P. lagopus* extract’s phenolic capacity and its main phytochemical components.

## Materials and methods

### Solvents and reagents

Phenolic standards, namely, catechin, catechol, syringic acid, chlorogenic acid, cinnamic acid, ellagic acid, kaempferol, ferulic acid, gallic acid, rutin, caffeic acid, naringenin, benzoic acid, o-coumaric acid, p-hydroxybenzoic acid, pyrogallol, p-coumaric acid, quercetin, quinol, rosmarinic acid, and vanillic acid were bought from Merck KGaA (Darmstadt, Germany). The purities of standards were up to 99%. The solvents used were of analytical grade, dimethylsulfoxide (DMSO; Alfa Aesar GmbH & Co KG, Massachusetts, United States), orthophosphoric acid (H_3_PO_4_), ethanol, methanol, and acetonitrile HPLC-grade (Fisher Scientific International, Inc., Hampton, New Hampshire, United States).

### Fungus isolation and identification

Tomato samples with root rot disease-like symptoms were gathered from El-Beheira Governorate, Egypt. The roots of the symptomatic plants were cut and washed with running tap water, then cleaned and sterilized several times before drying in laminar flow. PDA media were used in the isolation and cultivation process. The fungus purification was performed with the hyphal tip procedure (Dhingra and Sinclair, 2017). According to the previous descriptions (Alexopoulos et al., 1996), the fungus was identified based on morphological and microscopic characteristics. Moreover, molecular identification processes were performed using ITS1 and ITS4 primers (Table 1). PCR reaction conditions were done as previously described (Heflish et al., 2021). The PCR product was electrophoresed on agarose gel, purified, and subjected to sequencing. The obtained nucleotide sequences were aligned using MEGA X software and compared to other related organisms using NCBI-BLAST. After that, the fungus sequence was subjected to the NCBI-GenBank submission portal to obtain the accession number.

**Table 1:** HPLC conditions and operations used to detect the phenolic and flavonoid compounds.

HPLC conditions	Phenolic compounds	Flavonoid compounds	References
Instrument	Agilent 1,260 Infinity HPLC is equipped with an Infinity II analytical Quaternary pump and a column Zorbax Eclipse plus C18 (100 mm × 4.6 mm i.d.) with a particle size of 3.5 μm (Agilent, Santa Clara, CA, United States)	Smart line (Knauer, Germany) equipped with a binary pump and a Zorbax Eclipse plus C18 (column 150 mm × 4.6 mm i.d.) with a particle size of 5 μm (Agilent Technologies, Santa Clara, CA, United States)	Al-Huqail et al. (2019); Abdelkhalek et al. (2020a); Ashmawy et al. (2020a)
Temperature of operation	30°C	35°C	
Separation elution gradient	A: HPLC grade water 0.2% H_3_PO_4_ (v/v) B: Methanol C: Acetonitrile Flow rate 1.0 ml/min	Methanol: H_2_O with 0.5% H_3_PO_4_ (50:50) Flow rate 0.7 ml/min	
Injection volume	5 μl	5 μl	
Detector	Variable wavelength detector (VWD) at *λ* = 284 nm	UV absorption at *λ* = 273 nm	

### Preparation of *Plantago lagopus* extract

*Plantago lagopus* plants, gathered from the northwest of Egypt, were left to dry at room temperature (25°C) for a week and crushed to a fine powder using a grinder mill (Moulinex AR1044, France). Approximately 100 g of the dried powder was left in 200 ml of 95% ethanol for 4 days (Abdelkhalek et al., 2021). The alcoholic mixture was filtered through Whatman filter paper No. 1, and the obtained extract was evaporated and concentrated by a rotary evaporator until all the ethanol was removed. The *P. lagopus* extract was reserved in a brown bottle at 4°C until use. In the *in vitro* experiment, the extract was diluted with DMSO to obtain different concentrations (μg/mL). In the *in vivo* experiment, the extract was prepared in 0.1% Tween® 80 (w/v) to get a 100 μg/ml concentration.

### Antioxidant activity of *Plantago lagopus* extract

The free radical scavenging activity was measured according to previously described methods (Shimada et al., 1992). A 3.94 mg of DPPH (1,1-diphenyl-2-picrylhydrazyl) was diluted to 0.1 ml in 100 ml of methanol. A 1 ml of diluted DPPH was mixed with 3 ml of each sample with different concentrations (250, 125, 62.5, 31.25, and 15.62 μg/ml). The mixture was well mixed by vigorous shaking and stored at 25°C for 30 min. Each combination’s absorbance value (AV) was spotted at 517 nm. $\mathrm{Inhibition of DPPH was calculated}\;\mathrm{as}\;I\%=\left\{\left(\mathrm{Ao}-\mathrm{As}\right)/\mathrm{Ao}\right\}\times 100$, where As is the sample AV and Ao is the control AV reaction (contains all reagents except for the sample).

### HPLC analysis of polyphenolic components from the plant extract

The polyphenolic components of ethanolic *P. lagopus* plant extract were determined using HPLC-Agilent. HPLC conditions and program properties are illustrated in Table 1.

### Quantification of HPLC-detected compounds

Quantification was performed by the standard external method. The standard stock solutions (1–25 mg/l). A quantitative report is generated by combining known data from a calibration standard and unknown data from the sample. The UV detector can quantify a minimum concentration of 0.1 g/ml.

### The antifungal activity of *Plantago lagopus* extract against root rot fungus *in vitro*

Using the food poisoning technique, *P. lagopus* extract was tested for its efficiency against the root rot pathogen (Kumar et al., 2008). Different concentrations of *P. lagopus* extract were prepared in final (2, 4, 6, 8, and 10) μg/mL by emerging in PDA plates (90 mm diameter) compared with fungicide (Fluconazole 2.5 μg/ml), and the negative control (PDA plates without any additives). Fungus discs were cut from the periphery of 6 days old cultures, inoculated in the center of the treated poured Petri dishes, and then incubated at 25°C for a week. Three replicates were used for each treatment. The efficacy of *P. lagopus* extract on the fungus linear growth was measured. The growth $\mathrm{inhibition was calculated}\;\mathrm{as}\;\left(\%\right)=\left[\left(C0-\mathrm{Tx}\right)/C0\right]\times 100$ (Dissanayake, 2014), where C0 represents the length of the fungus growth on the control PDA plates and Tx represents the fungus growth in the plant extract treatment.

### Greenhouse experimental design and growth parameters assessment

The ability of *P. lagopus* extract to reduce *R. solani* and promote tomato growth was tested in a pot trial under greenhouse climate conditions (28 ± 2°C; 75 ± 5%; 14 h light/10 h dark). Each pot (20 × 13.2 × 12.2 cm) was filled with sterilized soil and planted with 4-week-old tomato seedlings of the Peto 86 variety. Five days after transplanting, the plant extract of *Plantago* was used with a 100 μg/ml concentration and added to the potting soil (20 ml/pot). *R. solani* inoculum was prepared by inoculating pre-sterilized autoclaved moistened barley grains (500 g) with 2 plugs of *R. solani* (0.5 cm in diameter) and incubating for 1 week at 25 ± 2°C. By the end of incubation, the barley inoculum was air-dried, milled to a fine powder, and applied to the pots (5 g inoculum/kg) where the inoculum was introduced close to the root pan and crown of the plant was going to be placed (Montealegre et al., 2010). The treatments were distributed in five replicates. (1). a protective treatment in which plant extract was added 2 days before *R. solani* inoculation, (2). a curative treatment in which *R. solani* was inoculated 2 days before plant extract application, (3). a *Rhizoctonia* treatment in which tomato plants were only inoculated with *R. solani*, and (4). a plant extract treatment in which tomato plants were only treated with *P. lagopus* extract. The last treatment was the control treatment, in which tomato plants were inoculated with PDA-free microorganisms. The plants were carefully uprooted after 1 month of transplanting for root rot severity screening according to a 0–5 scale (Abdeljalil et al., 2016). The data were calculated as disease index percentage $\left(\mathrm{DI}\right)\%=\left[\left(\mathrm{Number of infected plants}\right)/\left(\mathrm{Total plants number}\right)\right]\times 100.$

The uprooted tomato plants were also used to measure the effectiveness of the *P. lagopus* extract on different tomato growth parameters, such as plant height (cm), root length (cm), shoot fresh and dry weight (g), root fresh and dry weight (g), and the total chlorophyll content (SPAD value). Furthermore, tomato leaf samples were collected from each treatment to study the activity of the defense-related genes and total phenolic components in response to different treatments under pot trials. Tomato leaves were collected 20 days of post-inoculation (dpi) with *R. solani*.

### Total phenolic content in tomato plants

Folin–Ciocalteu (FC) method was used to measure the TPC values of tomato extracts. Tomato extract (100 μl, 1 mg/ml) was combined with 750 μl of FC (diluted to 1:10 in water). The mixture was left at 25°C for 5 min, then 750 μl of sodium carbonate was added to the mix and gently shaken. After 90 min, the absorbance value of the blend was measured at 725 nm. A calibration curve was created using a standard reference (a gallic acid concentration range of 0.01 to 0.05 mg/ml). As described previously (Velioglu et al., 1998), TPC was determined as gallic acid equivalents (μg GAE/100 g extract).

### Defense-related genes

#### RNA extraction and cDNA synthesis

Plant total RNA was manually extracted from about 100 mg of tomato leaves collected at 20 dpi using a modified guanidium isothiocyanate (GITC) method (Abdelkhalek and Sanan-Mishra, 2019). SPECTROstar Nano was used to determine the purity and concentration of extracted RNA (BMG Labtech, Ortenberg, Germany). As previously described (AbdEl-Rahim et al., 2010; Abdelkhalek et al., 2019), the isolated RNA was utilized to generate cDNA using a reverse transcriptase (RT) enzyme. In an Eppendorf cycler (Hamburg, Germany), the RT reaction was conducted at 42°C for 1 h and then deactivated at 80°C for 5 min. The RT-PCR product was stored at −20°C until employed as a qRT-PCR template.

#### qRT-PCR assay and data analysis

The expression levels of three tomato pathogenesis-related genes (*PR-1*, *PR-2*, and *PR-3*) and one polyphenolic gene (*CHS*) were analyzed using the qPCR and normalized to the reference *β*-actin gene. Primer nucleotide sequences used in this investigation are listed in Table 2. For each biological sample, the experiments were repeated three times. As previously described (Abdelkhalek et al., 2018), qRT-PCR was carried out using a QIAGEN Rotor-Gene 6,000 (ABI System, United States) with Thermo SYBR Green Mix (Foster, CA, USA). The relative expression level of the tested gene was calculated according to the 2^−ΔΔCT^ method (Livak and Schmittgen, 2001).

**Table 2:** Primers used in this investigation.

Gene	Abbreviation	Nucleotide sequences
Internal transcribed spacer	ITS	ITS1-TCCG TAG GTG AACCT GCGG
		ITS4-TCCT CCGC TTAT TGA TATGC
Pathogenesis related protein-1	*PR-1*	For-GT TCCT CCT TGC CAC CTTC
		Rev-TATGC ACCC CCA GC ATAGTT
Endoglucanase	*PR-2*	For-TATA GCC GTTG GAA ACG AAG
		Rev-CAACT TGC CATC AC ATTCTG
Chitinase	*PR-3*	For-ATGG AGCA TTG TGCC CTAAC
		Rev-TCCTA CCA ACA TCAC CAC CA
Chalcone synthase	*CHS*	For-CAC CGTG GAG GAG TA TC GTA AGGC
		Rev-TGA TCA ACA CAGTT GGAA GGCG
*β*-actin	*β*-actin	For-TGG CAT ACAA AGAC AGGA CAG CCT
		Rev-ACT CA ATC CCA AGGC CA ACA GAGA

### Statistical analysis

The data were analyzed using CoStat software, and significant differences were estimated using Tukey’s honest significant differences technique (H.S.D.) at a *p* ≤ 0.05, with standard deviation (SD) presented as a column bar or values. Upregulation of a gene means that the relative expression levels are greater than 1, whereas downregulation means values less than 1.

## Results

### Identification of *Rhizoctonia solani* fungus

**Figure 1:**
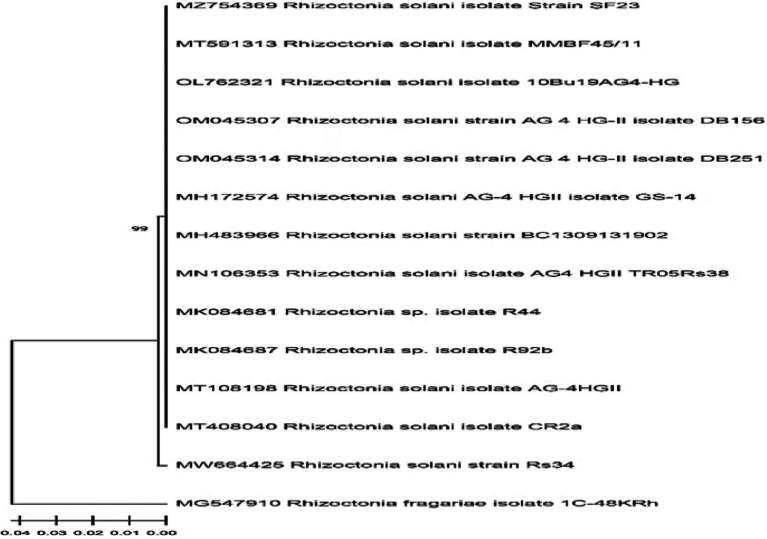
The phylogenetic cladogram shows the relationship of the *Rhizoctonia solani* Rs34 among closely related *R. solani* sequences. The GenBank alignment was based on partial inter transcripted spacer region (ITS) sequences.

Following accepted taxonomic phenotypic standards, the morphological study of the isolated *Rhizoctonia* isolate from tomato plant roots was compatible with *Rhizoctonia* genus characteristics. The amplified ITS fragment partial sequence was obtained, then submitted to GenBank, and was confirmed to be *Rhizoctonia solani* strain Rs34 and assigned the accession number MW664425. The alignment of the received sequence of *R. solani* Rs34 with the other sequences downloaded from the GenBank database indicated that the genetic homogeneity nearest to 99% of the ITS partial sequence was with *R. solani* (MT408040, MT108198, and MZ754369) and isolates of *Rhizoctonia* sp. (MK084681 and MN106353) Figure 1.

### Effect of *Plantago lagopus* extract on mycelial growth of *Rhizoctonia solani*

The growth reductions of *R. solani* in response to the tested plant extracts are presented in Table 3. Radial growth of *R. solani* decreased significantly with an increase in the concentration of plant extracts from 2 to 10 μg/ml. It was found from the results that the extract of *Plantago lagopus* caused 100% inhibition of *R. solani* mycelial growth at 10 μg/ml. At the same time, there was no effect on *R. solani* mycelial growth at the dose of 2 μg/ml compared with antifungal fungicide (Fluconazole, 2.5 μg/ml).

**Table 3:** *In vitro* growth inhibition (%) of *Rhizoctonia solani* in response to *Plantago lagopus* extract.

Treatment (**μ**g/mL)	Growth inhibition %
Negative control	0.00
2	0.00
4	38.07
6	88.56
8	89.78
10	100.00
Fungicide (Fluconazole, 2.5 μg/ml)	100.00

### Effect of *Plantago lagopus* extract on *Rhizoctonia solani* root rot disease

**Figure 2:**
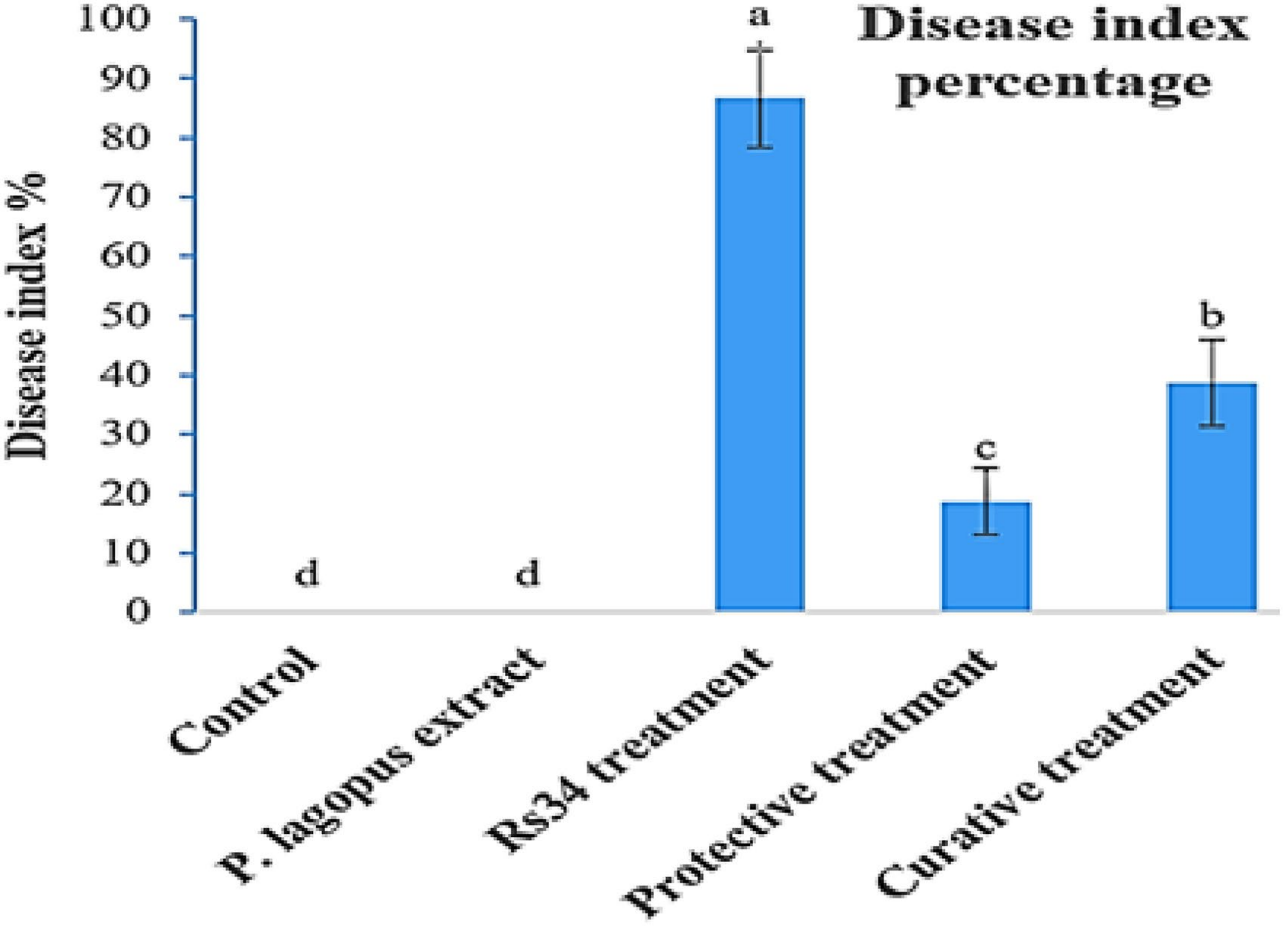
Effect of *Plantago lagopus* extract on disease index (DI%) of tomato root rot caused by *Rhizoctonia solani* under greenhouse conditions. The different letters **(a–d)** represent significant differences.

The extract of *P. lagopus* was tested for its efficacy on *R. solani* under greenhouse growth conditions. The disease index (DI%) was estimated according to the root browning symptoms and their extent levels (using a 0–5 scale) based on each treatment (Figure 2). The plant extract substantially reduced the DI% compared to the untreated control. *P. lagopus* extract application before inoculation with *R. solani* (protective treatment) showed a significant reduction of the percentage of disease index (18.66%), followed by the curative treatment (38.66%; Figure 2). DI% was 86.66% in the *Rhizoctonia* treatment and 0.0% in the control and plant extract treatments.

### Effect of *Plantago lagopus* extract on tomato growth parameters

**Figure 3:**
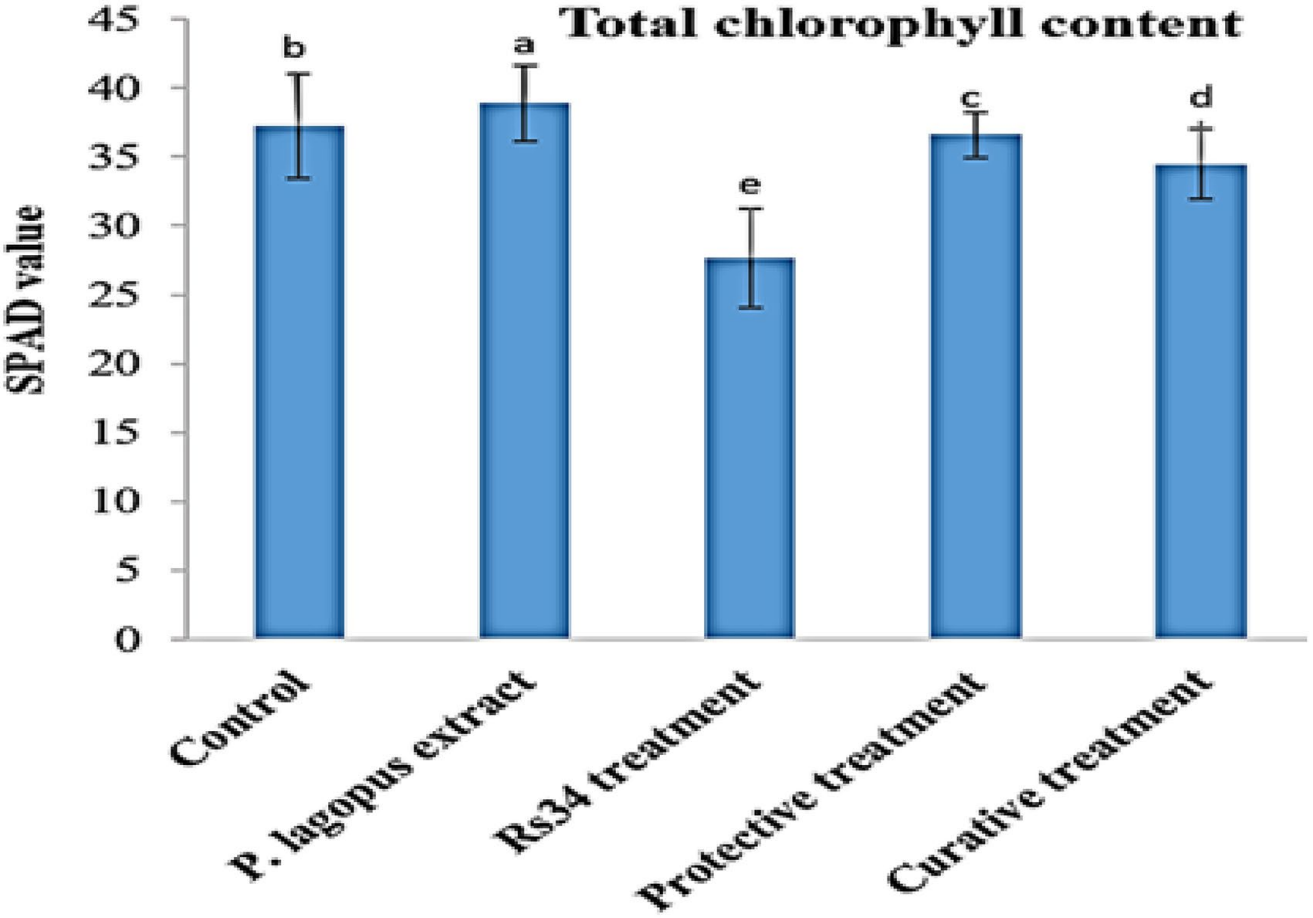
Effect of all treatments on total chlorophyll content (SPAD value) in tomato plants as affected by root rot disease under greenhouse conditions. The different letters **(a–e)** represent significant differences.

In the greenhouse experiment, both protective and curative treatments of *P. lagopus* extract showed a significant enhancement (*p* ≤ 0.05) in the growth of tomato plants (Table 4). Moreover, the influence on the height was substantial due to the plant extract treatment. It was recorded that plant height was 36.60 cm in plant extract treatment, followed by protective treatment, which recorded 36.20 cm. Compared to *Rhizoctonia* treatment (5.2 cm), the plant extract and protective and curative treatments significantly increased root length by 21.30, 21.10, and 20.70 cm, respectively (Table 4). The plant extract, protective, and curative treatments increased shoot-fresh weight (14.12, 13.78, and 12.24 g, respectively) and root-fresh weight (5.96, 5.70, and 5.32 g, respectively) more than the *Rhizoctonia* or control treatments. In addition, non-significant results in all treatments were noticed for the dry shoot weights compared to control. The root dry weight of tomato plants changed after being treated with the plant extract, protective, and curative treatments compared with control (Table 4). The plant extract treatment was more effective in increasing chlorophyll content (38.88, SPAD value), followed by the protective and curative treatments, with significant SPAD values of 36.58 and 34.46, respectively, compared with the control (37.22) and *Rhizoctonia* treatment (27.64) Figure 3.

**Table 4:** Effect of treatment with *Plantago lagopus* extract on growth parameters of tomato plants.

Treatments	Plant height (cm)	Root length (cm)	Shoot fresh weight (g)	Root fresh weight (g)	Shoot dry weight (g)	Root dry weight (g)
Control	34.40 ± 5.31 a	08.90 ± 2.30 b	07.15 ± 2.49 b	1.78 ± 0.67 b	3.20 ± 0.73	1.20 ± 0.22 b
Plant Extract	36.60 ± 2.07 a	21.30 ± 3.17 a	14.12 ± 2.64 a	5.96 ± 1.35 a	3.38 ± 0.34	2.30 ± 0.12 a
Curative	35.00 ± 5.87 a	20.70 ± 9.05 a	12.24 ± 4.38 a	5.32 ± 1.54 a	3.20 ± 0.49	2.42 ± 0.16 a
Protective	36.20 ± 3.70 a	21.10 ± 2.24 a	13.78 ± 2.86 a	5.70 ± 0.44 a	3.10 ± 0.29	2.40 ± 0.20 a
*Rhizoctonia*	25.40 ± 8.79 b	05.20 ± 0.57 b	06.12 ± 3.01 b	1.65 ± 0.57 b	2.82 ± 0.69	1.28 ± 0.30 b
*p*-Value	0.0287	<0.0001	0.0009	<0.0001	0.5910^ns^	<0.0001

### Transcriptional levels of defense-related genes

**Figure 4:**
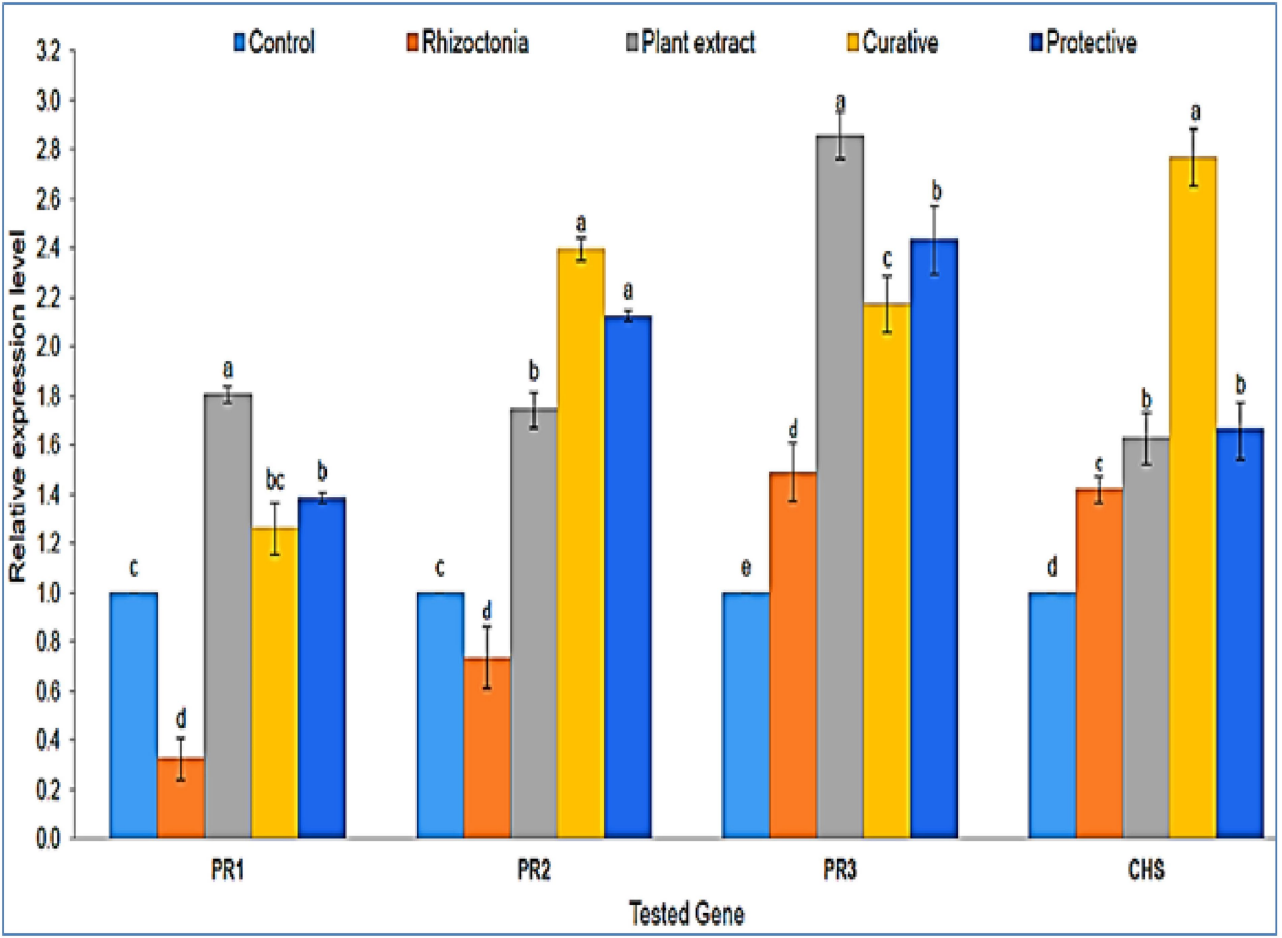
A histogram shows the relative expression levels of the four genes *PR-1*, *PR-2*, *PR-3*, and *CHS* at 20 dpi of plant extract treatments (100 μg/ml) in different treatments compared with control. The different letters **(a–e)** represent significant differences.

At 20 dpi, the inhibitory effects and relative transcriptional levels of four defense-related genes (*PR-1*, *PR-2*, *PR-3*, and *CHS*) were assessed. The antifungal activity of *P. lagopus* against fungal infection was validated by qPCR data, which showed a considerable elevation of defense genes inside the treated plants (Figure 4). Compared to the controls, a significant upregulation of *PR-1* was observed in all plant extract treatments, while non-treated plants showed a downregulation. The highest relative expression level (1.81-fold) was reported in plant extract treatment, followed by protective and curative treatments with expression levels of 1.39- and 1.26-fold, respectively. The expression level of *PR-2* showed upregulation in all plants treated with plant extract (Figure 4). The most outstanding transcriptional level (2.39-fold) was reported in curative treatment, followed by protective treatment with a relative transcriptional level of 2.12-fold compared to the control. On the other hand, plants in *Rhizoctonia* treatment showed a downregulation with a relative transcriptional level of 0.74-fold compared to the control (Figure 4).

Regarding *PR-3* expression, a significant upregulation was shown with all treatments compared to untreated plants (Figure 4). The highest expression level (2.85-fold) was observed with plant extract-only treatment, followed by protective, curative, and non-treated with relative transcriptional levels of 2.43-, 2.17-, and 1.49-fold, respectively. The *PR-3* gene produces the chitinase enzyme, which hydrolyzes the chitin and prevents plants from fungi invasions (Abdelkhalek et al., 2021). The *PR-3* gene was upregulated in tomato plants infected with *Rhizoctonia* or treated with *P. lagopus* or the curative or protective treatments in the current investigation. *P. lagopus*-treated plants had the highest relative expression. The *CHS* gene showed an upregulated expression level in all treatments compared to untreated plants (control). The expression level of curative treatment exhibited the greatest level (2.77-fold), followed by protective, plant extract, and non-treated plants treatments with 1.66-, 1.62-, and 1.42-fold higher expression levels than control, respectively (Figure 4).

### TPC accumulation in treated tomato plants

The phenolic concentration in the protective treatment was about 10-fold higher than in control, while in *P. lagopus* extract or *R. solani* treatments, it was 5.97- and 5.82-fold higher (Table 5). Additionally, in the curative treatment, the phenolic concentration was lower than those found in all treatments except the control (0.039 μg GAE/100 g) but still higher, by about 4.1-fold, than control plants.

**Table 5:** Total phenolic components activities of all treatments used in this study.

Treatment	Total Phenolic compounds (**μ**g GAE/100 g) ± SD
Control	0.039 ± 0.009 d
Plant extract	0.233 ± 0.010 b
Protective	0.388 ± 0.008 a
Curative	0.160 ± 0.005 c
*Rhizoctonia*	0.227 ± 0.020 b
*P*-value	<0.0001

### Antioxidant activity of the *Plantago lagopus* extract

The DPPH method was used to determine the antioxidant potential of *P. lagopus* extract to act as a free radical scavenger. The antioxidant activity value of the extract was 80.23 μg/ml compared with the control ascorbic acid (5.04 μg/ml).

### HPLC analysis of *Plantago lagopus* extract

**Figure 5:**
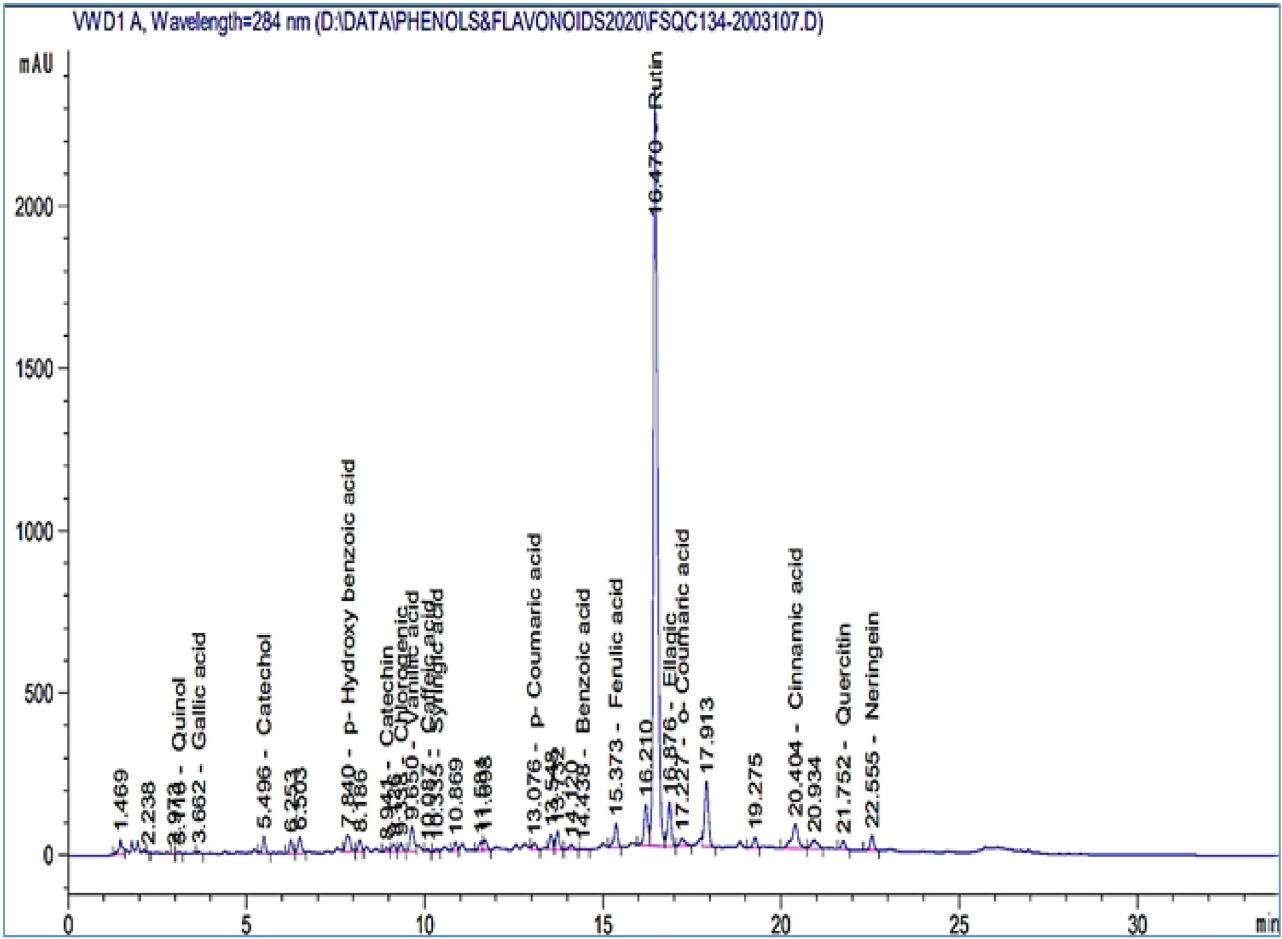
HPLC chromatograms of the phytochemical compounds identified in *Plantago lagopus* extract.

HPLC was used to identify the *P. lagopus* extract compounds. The identified chemical compounds are shown in Figure 5 and Table 6. The main compounds were determined as mg/kg: rutin (74206.3), naringenin (2388.74), quercetin (1249.13), *p*-hydroxybenzoic acid (1035.87), ellagic acid (798.47), vanillic acid (752.55), catechol (648.89), cinnamic acid (332.51), ferulic acid (296.32), benzoic acid (295.95), and chlorogenic acid (116.63).

**Table 6:** Phenolic and flavonoid components identified in *Plantago lagopus* extract.

Components	RT (min.)	Amount (mg/kg)
Quinol	3.116	91.3
Gallic acid	3.662	23.68
Catechol	5.496	648.89
*p*-Hydroxy benzoic acid	7.840	1035.87
Catechin	8.941	27.01
Chlorogenic acid	9.338	116.63
Vanillic acid	9.650	752.55
Caffeic acid	10.087	20.48
Syringic acid	10.335	42.41
*p*-Coumaric acid	13.076	65.19
Benzoic acid	14.438	295.95
Ferulic acid	15.373	296.32
Rutin	16.470	74206.3
Ellagic acid	16.876	798.47
*o*-Coumaric acid	17.227	74.06
Cinnamic acid	20.404	332.51
Quercetin	21.752	1249.13
Naringenin	22.555	2388.74

## Discussion

*Rhizoctonia solani* is a widespread fungus disease in agricultural soils and a major factor impacting the germination of many plant seedlings causing damping-off symptoms (El-Kazzaz et al., 2022). Chemical fungicides are routinely and effectively used to control *Rhizoctonia* root-rot. However, their application in the field might not always be desired. Considering the disadvantages of chemical control of plant diseases, using plant extracts to combat plant diseases is gaining prominence (Abdelkhalek et al., 2020b). Many plant materials, like plant extracts, essential oils, gum, resins, etc., have shown biological activity *in vitro* and *in vivo* and are used as biofungicides. In the current study, the antifungal activities of the ethanolic extract of *P. lagopus* against *R. solani* were evaluated on tomato plants under greenhouse conditions for the first time. Moreover, its effect on the tomato growth parameters, chlorophyll content, phenolic content, and defense-related gene expression was also assessed. Furthermore, the phytochemical components of the *P. lagopus* extract were identified using HPLC.

The morphological study of *Rhizoctonia* isolates from tomato plant roots was compatible with *Rhizoctonia* genus characteristics as it is an anamorphic mycelial septate fungus that did not produce asexual spores (Heflish et al., 2021). The ITS-PCR reaction confirmed the morphological identification to be *R. solani* strain Rs34. As stated before, pesticides, biological control, and azoles fungicides effectively reduce agricultural losses caused by fungal diseases (Hof, 2001; Singh et al., 2016). Our results revealed that the growth of *R. solani* was reduced significantly in response to the *Plantago lagopus* extract from 2 to 10 μg/ml compared to Fluconazole (positive control). While Fluconazole had the better results in our study, it is often used in commercial rapid antifungal susceptibility testing (Rex et al., 1993). It is one of the azole groups that had higher minimal inhibitory concentrations (MICs) against most *Fusarium* species (Al-Hatmi et al., 2015), and 90% of *Candida albicans* isolates had Fluconazole MICs of less than 1 μg/ml (Rex et al., 1995). In some conditions, Fluconazole has been proven ineffective against yeasts other than *C. albicans* (Rex et al., 1995).

The antifungal effect of the *P. lagopus* extract *in vitro* against *R. solani* in this study agreed with our recent research results. The ethanolic *Coccoloba uvifera* extract inhibited the development of *R. solani*, *Botrytis cinerea*, and *Fusarium culmorum* by 64.4, 100, and 38.5%, respectively (Ashmawy et al., 2020b). Also, the results are consistent with those documented by different authors (Deena and Thoppil, 2000; Valarini et al., 2003). They discovered that extracts of lantana, garlic, eucalyptus, and lemongrass have an antifungal effect on soil fungi mycelial growth. Besides, the n-hexane extract of *Eucalyptus camaldulensis* demonstrated the same high antifungal property against *F. culmorum* and *R. solani*, especially at 3% (Salem et al., 2019). Similarly, wood samples treated with *Acacia saligna* water extract inhibited the growth of *F. culmorum* and *R. solani* mycelium (Al-Huqail et al., 2019). Meanwhile, *Plantago* spp. such as *P. major* had antifungal action against all phytopathogenic fungi examined with 2000 μg/ml extract. The highest growth inhibition (32.2%) was observed against *Phytophthora cinnamomi*, followed by *Colletotrichum gloeosporioides* (25.7%), *C. godetiae,* and *C. nymphaeae* (21.1%; Ferreira and Oliveira, 2020). The ethyl acetate fraction of *Plantago* sp. was shown to be the most potent *in vitro* against *Staphylococcus aureus* and *Pseudomonas aeruginosa* bacteria (Karima et al., 2015), while *Klebsiella pneumoniae*, *Proteus mirabilis*, and *Salmonella typhimurium* had the lowest antibacterial activity (inhibition zone, 16.7 and 13.3 mm; Karima et al., 2015).

Under greenhouse growth conditions, the extract of *P. lagopus* was tested for its efficacy on *R. solani* and revealed a significant reduction in disease symptoms. The same results could be concluded from the study of Al-Askar and Rashad (Al-Askar and Rashad, 2010). They observed the efficacy of clove extract on pea root-rot disease incidence in a greenhouse experiment as the clove extract at 4% concentration, and a chemical fungicide showed a considerable improvement in the proportion of plants that survived (40 and 48%, respectively) and a significant decrease in disease incidence. Furthermore, our findings were consistent with a previous field experiment (Abd-El-Khair and El-Gamal Nadia, 2011). They reported that using chilli, lantana, and lemongrass extracts revealed an incidence reduction of *R. solani* damping-off disease. Even though some researchers who focused only on aqueous extracts found that these extracts had antifungal efficacy against specific fungi (Bhardwaj, 2012), other studies have compared the antimicrobial activities of alcoholic and aqueous extracts and noticed that the alcoholic is better than aqueous extracts (Ambikapathy et al., 2011; Ashraf et al., 2011; Behbahani et al., 2013; Jat and Agalave, 2013; Moorthy et al., 2013). In the greenhouse experiment, both protective and curative treatments of *P. lagopus* extract showed a significant enhancement (*p* ≤ 0.05) in the growth of tomato plants. Several studies had the same effective results that *Moringa oleifera* leaf extract increased tomato growth and yield (Culver et al., 2012), common bean growth and yield (Mvumi et al., 2013), and some *Eruca vesicaria* growth parameters and photosynthesis rate (Abdalla, 2014). Blueberry fruits, red grape, and hawthorn leaf extracts have improved maize growth, total chlorophyll content, and roots biomass (Ertani et al., 2016). Also, the secondary metabolites, including a wide range of compounds such as flavonoids, terpenoids, alkaloids, and phenolics, have bioactivity as stimulants or inhibitors of plant growth, as reported by many authors (James and Dubery, 2011; Singh et al., 2012; Biradar and Rachetti, 2013).

The relative transcriptional levels of four defense-related genes (*PR-1*, *PR-2*, *PR-3*, and *CHS*) were assessed at 20 dpi, which showed a considerable regulation of the four defense genes inside the treated plants. *PR-1* is a crucial regulator of SAR and may serve as a marker for early plant defense responses (Hoegen et al., 2002), and its importance in plant immunity has been recognized for over two decades. The accumulation and expression of *PR-1*, a SA marker gene, are linked to the activation of SA in response to pathogens (D’Maris Amick Dempsey et al., 2011; Abo-Zaid et al., 2021). Compared to the controls, a significant upregulation of *PR-1* was observed in all plant extract treatments, while non-treated plants showed a downregulation. This is consistent with previous findings of many authors (Su et al., 2013; Chandrasekaran and Chun, 2016), indicating that increased PR expression aids resistance against hemibiotrophic and necrotrophic pathogen infections. As a result, it is possible that the *P. lagopus* can modify the plant’s response, increase resistance, and prevent *R. solani* from suppressing defense genes. As a result of sterol extraction from fungal cell membranes, sterol-binding PR1 proteins limit fungal cell proliferation (Schneiter and Di Pietro, 2013), while PR2-1,3-glucanases, combined with *PR-3*-chitinases, lysis the fungus wall (Balasubramanian et al., 2012). The transcriptional levels of *PR-2* were upregulated in the protective and curative treatments and downregulated in *Rhizoctonia* treatment compared to the control. Several reports have confirmed that *PR-2* proteins are engaged in various physiological plant defense processes primarily induced by SAR inducers like SA (Abdelkhalek, 2019). The fungal secondary metabolites may have boosted *PR-2* activity in *P. lagopus* extract-treated plants (Druzhinina et al., 2011). This conclusion aligns with recent research that shows microbial elicitors enhance *PR-2* mRNA in plants (Roylawar et al., 2015). Increased *PR-2* activity in the cell wall has been shown to enhance the number of oligosaccharides produced, which elicits plant defense systems.

The *PR-3* gene was upregulated in tomato plants infected with *Rhizoctonia* or treated with *P. lagopus* or the curative or protective treatments in the current investigation. *P. lagopus* -treated plants had the highest relative expression. The *PR-3* gene produces the chitinase enzyme, which hydrolyzes the chitin and prevents plants from fungi invasions (Abdelkhalek et al., 2021). The findings show that the *PR-3* gene helps to strengthen plant resistance to fungal attacks. The application of *P. lagopus* increases the activation of multiple defensin genes, including *PR-3*, in leaf tissue, resulting in increased pathogen resistance (Heflish et al., 2021). The *CHS* gene showed an upregulated expression level in all treatments compared to control. *CHS* is a required precursor or first enzyme in plant flavonoids biosynthesis, converting *p*-coumaroyl CoA to naringenin chalcones (Abdelkhalek et al., 2020b). Surprisingly, *P. lagopus* extract, curative and protective treatments, resulted in the highest induction of *CHS*, which is necessary for flavonoid production (Marais et al., 2006; Abdelkhalek et al., 2021). In previous investigations, *CHS* overexpression was observed to produce large quantities of flavonoids with broad antifungal action against various plant pathogens (Martínez et al., 2017; Abdelkhalek et al., 2020a). As a result, treating tomato plants with *P. lagopus* as a protective or curative treatment may increase the quantity of flavonoid compounds. As a result, we believe that the *P. lagopus* extract contains elicitor metabolite chemicals that can activate SAR and boost plant resistance to fungal infection. Following pathogen exposure, the activation of defense-related genes has been described in numerous plant-pathogen interactions in the primed state (Ahn et al., 2007; Niu et al., 2011). This is also apparent in ISR against hemibiotrophic and necrotrophic diseases, as evidenced by *Harpophora oryzae*-primed defense genes in the rice-*Magnaporthe oryzae* interaction (Su et al., 2013) and *B. subtilis*-induced PR genes in tomato challenged with *Pectobacterium carotovorum* (Chandrasekaran and Chun, 2016). However, *P. lagopus* could be used to combat *R. solani* infections as a biocontrol agent. However, further research is needed for future field uses.

Plants treated with plant extract result in the buildup of phenolic components, linked to reactive metabolic defense against infectious pathogens. Various studies reported that fungal or bacterial pathogen infections caused a potent increase in total phenolic content (Martin et al., 2009; Petkovsek et al., 2011; Rusjan et al., 2012). Consequently, polyphenolic compounds that accumulate in plant extract-treated plants potentially act as proton donors, reducing oxidative injury to root cells during pathogen infections (Singh et al., 2011). Therefore, the increase in TPC as a plant response to the plant extract application in protective treatment alone would be associated with protection against plant pathogens in tomato plants. These differences in TPC are caused by the phenylpropanoid pathway regulation, as cited (Mikulic-Petkovsek et al., 2014). Our results coincided with numerous studies showing that *Plantago* species had antioxidant activity. Few studies showed high antioxidant activity for *Plantago* spp. (Çoban et al., 2003; Gálvez et al., 2005; Beara et al., 2009), and P. major (Samuelsen, 2000; Chiang et al., 2002). In addition, a study on the antioxidant activity of *P. lanceolate* in different extracts showed that ethanolic extracts had the highest DPPH free radical scavenging capacity (Miser-Salihoglu et al., 2013).

The identified chemical compounds in HPLC analysis revealed the existence of many polyphenolic compounds such as rutin, naringenin, quercetin, *p*-hydroxybenzoic acid, ellagic acid, vanillic acid, catechol, cinnamic acid, ferulic acid, benzoic acid, and chlorogenic acid. Many polyphenols have antimicrobial, antiviral, and antioxidant properties, including ferulic acid, quercetin, ellagic acid, chlorogenic acid, catechins, gallic acid, caffeic acid, and myricetin (Shaygannia et al., 2016; Mani et al., 2020). According to our findings, the polyphenolic compounds may act as elicitor molecules and play essential roles in SAR. *P. lanceolata* plants are rich in polyphenolic compounds such as syringic acid, cinnamic acid, resveratrol, caffeic acid, ferulic acid, rutin, and quercetin. In contrast, in *P. major* plants, more flavonoids, iridoid glycosides, triterpene acids, caffeic acid, chlorogenic acid, vanillic acid, and *p*-coumaric acid were found in their HPLC-UV analysis (Samuelsen, 2000). Our earlier research has revealed a broad spectrum of phenolic chemicals responsible for various medicinal plants’ antioxidant and antibacterial activities (Mohamed et al., 2020). However, *P. lagopus* could be used to combat *R. solani* infections as a biocontrol agent. However, further research is needed for future field uses.

## Conclusion

In this study, the application of the *P. lagopus* extract (100 μg/ml) boosted tomato plant growth, inducing systemic resistance and decreasing root rot disease caused by *R. solani* fungus. Based on our findings, the reduction in disease incidence was seen in *P. lagopus*-treated plants compared to control plants, along with upregulation increases of *PR-1* and *PR-2* and a significant increase in the transcriptional levels of *PR-3* and *CHS* genes at 20 dpi. *P. lagopus* extract-HPLC analysis indicated that the main compounds were rutin, naringenin, quercetin, *p*-hydroxybenzoic acid, ellagic acid, vanillic acid, catechol, cinnamic acid, ferulic acid, benzoic acid, and chlorogenic acid. According to our results, the protective treatment best reduces root rot disease. Furthermore, our study provides the basis for additional investigation of plant extract antifungal properties to limit the usage of fungicides, which pose many risks to human health and the environment.

## Data availability statement

The original contributions presented in the study are included in the article/supplementary material, further inquiries can be directed to the corresponding author.

## Author contributions

SB, AA, MS, and AH designed the research and wrote the manuscript. AA, SS, MS, ME, and SB performed the experiments. AA-A, AB, FA, ME, and EH helped with editing and provided suggestions for the experiments. AA, AH, MS, and SB analyzed the data. All authors contributed to the article and approved the submitted version.

## Funding

This research was financially supported by the Researchers Supporting Project number (RSP2022R505), King Saud University, Riyadh, Saudi Arabia.

## Conflict of interest

The authors declare that the research was conducted in the absence of any commercial or financial relationships that could be construed as a potential conflict of interest.

## Publisher’s note

All claims expressed in this article are solely those of the authors and do not necessarily represent those of their affiliated organizations, or those of the publisher, the editors and the reviewers. Any product that may be evaluated in this article, or claim that may be made by its manufacturer, is not guaranteed or endorsed by the publisher.

## References

[ref1] Abdalla M. M. (2014). Boosting the growth of rocket plants in response to the application of *Moringa oleifera* extracts as a biostimulant. Life Sci. J. 11, 1113–1121.

[ref2] Abdeljalil N. O.-B. Vallance J. Gerbore J. Bruez E. Martins G. Rey P.. (2016). Biocontrol of *Rhizoctonia* root rot in tomato and enhancement of plant growth using rhizobacteria naturally associated to tomato. J. Plant Pathol. Microbiol. 7, 1–8. doi: 10.4172/2157-7471.1000356

[ref3] Abd-El-Khair H. El-Gamal Nadia G. (2011). Effects of aqueous extracts of some plant species against *Fusarium solani* and *Rhizoctonia solani* in *Phaseolus vulgaris* plants. Arch. Phytopathol. Plant Prot. 44, 1–16. doi: 10.1080/03235400802678436

[ref4] Abdelkhalek A. (2019). Expression of tomato pathogenesis related genes in response to Tobacco mosaic virus. J. Anim. Plant Sci. 29, 1596–1602.

[ref5] Abdelkhalek A. Al-Askar A. A. Alsubaie M. M. Behiry S. I. (2021). First report of protective activity of *Paronychia argentea* extract against tobacco mosaic virus infection. Plan. Theory 10, 2435. doi: 10.3390/plants10112435, PMID: 34834798 PMC8620274

[ref6] Abdelkhalek A. Al-Askar A. A. Behiry S. I. (2020a). *Bacillus licheniformis* strain POT1 mediated polyphenol biosynthetic pathways genes activation and systemic resistance in potato plants against Alfalfa mosaic virus. Sci. Rep. 10, 1–16. doi: 10.1038/s41598-020-72676-2 32999301 PMC7527447

[ref7] Abdelkhalek A. Dessoky E. S. Hafez E. (2018). Polyphenolic genes expression pattern and their role in viral resistance in tomato plant infected with Tobacco mosaic virus. Biosci. Res. 14, 3349–3356.

[ref8] Abdelkhalek A. Ismail I. A. I. A. Dessoky E. S. E. S. El-Hallous E. I. E. I. Hafez E. (2019). A tomato kinesin-like protein is associated with Tobacco mosaic virus infection. Biotechnol. Biotechnol. Equip. 33, 1424–1433. doi: 10.1080/13102818.2019.1673207

[ref9] Abdelkhalek A. Salem M. Z. M. Hafez E. Behiry S. I. Qari S. H. (2020b). The phytochemical, antifungal, and first report of the antiviral properties of Egyptian *Haplophyllum tuberculatum* extract. Biology 9, 248. doi: 10.3390/biology9090248 PMC7565794 32854351

[ref10] Abdelkhalek A. Sanan-Mishra N. (2019). Differential expression profiles of tomato miRNAs induced by tobacco mosaic virus. J. Agric. Sci. Technol. 21, 475–485.

[ref11] AbdEl-Rahim W. M. Khalil W. K. B. Eshak M. G. (2010). Evaluation of the gene expression changes in Nile tilapia (*Oreochromis niloticus*) as affected by the bio-removal of toxic textile dyes from aqueous solution in small-scale bioreactor. Environmentalist 30, 242–253. doi: 10.1007/s10669-010-9268-7

[ref12] Abo-Zaid G. Abdelkhalek A. Matar S. Darwish M. Abdel-Gayed M. (2021). Application of bio-friendly formulations of Chitinase-producing *Streptomyces cellulosae* Actino 48 for controlling Peanut soil-borne diseases caused by *Sclerotium rolfsii*. J. Fungi 7, 167. doi: 10.3390/jof7030167, PMID: 33669115 PMC7996487

[ref13] Ahn I.-P. Lee S.-W. Suh S.-C. (2007). Rhizobacteria-induced priming in Arabidopsis is dependent on ethylene, jasmonic acid, and NPR1. Mol. Plant-Microbe Interact. 20, 759–768. doi: 10.1094/MPMI-20-7-0759, PMID: 17601164

[ref14] Al-Askar A. A. Rashad Y. M. (2010). Efficacy of some plant extracts against *Rhizoctonia solani* on pea. J. Plant Prot. Res. 50, 239–243. doi: 10.2478/v10045-010-0042-0

[ref15] Alexopoulos C. J. Mims C. W. Blackwell M. (1996). Introductory Mycology. New York, NY: John Wiley and Sons.

[ref16] Al-Hatmi A. M. S. van Diepeningen A. D. Curfs-Breuker I. de Hoog G. S. Meis J. F. (2015). Specific antifungal susceptibility profiles of opportunists in the *Fusarium fujikuroi* complex. J. Antimicrob. Chemother. 70, 1068–1071. doi: 10.1093/jac/dku505, PMID: 25538167

[ref17] Al-Huqail A. A. Behiry S. I. Salem M. Z. M. Ali H. M. Siddiqui M. H. Salem A. Z. M. (2019). Antifungal, antibacterial, and antioxidant activities of *Acacia saligna* (Labill.) HL Wendl. flower extract: HPLC analysis of phenolic and flavonoid compounds. Molecules 24, 700. doi: 10.3390/molecules24040700, PMID: 30781352 PMC6412425

[ref18] Ambikapathy V. Gomathi S. Panneerselvam A. (2011). Effect of antifungal activity of some medicinal plants against *Pythium debaryanum* (Hesse). Asian J Plant Sci Res 1, 131–134.

[ref19] Ashmawy N. A. Behiry S. I. Al-Huqail A. A. Ali H. M. Salem M. Z. M. (2020a). Bioactivity of selected phenolic acids and hexane extracts from *Bougainvilla spectabilis* and *Citharexylum spinosum* on the growth of *Pectobacterium carotovorum* and *Dickeya solani* bacteria: an opportunity to save the environment. Processes 8, 482. doi: 10.3390/pr8040482

[ref20] Ashmawy N. A. Salem M. Z. M. El Shanhorey N. Al-Huqail A. Ali H. M. Behiry S. I. (2020b). Eco-friendly wood-biofungicidal and antibacterial activities of various *Coccoloba uvifera* L. leaf extracts: HPLC analysis of phenolic and flavonoid compounds. Bioresources 15, 4165–4187. doi: 10.15376/biores.15.2.4165-4187

[ref21] Ashraf Z. Muhammad A. Imran M. Tareq A. H. (2011). In vitro antibacterial and antifungal activity of methanol, chloroform and aqueous extracts of *Origanum vulgare* and their comparative analysis. Int. J. Org. Chem. 1, 257–261. doi: 10.4236/ijoc.2011.14037

[ref22] Balasubramanian V. Vashisht D. Cletus J. Sakthivel N. (2012). Plant β-1, 3-glucanases: their biological functions and transgenic expression against phytopathogenic fungi. Biotechnol. Lett. 34, 1983–1990. doi: 10.1007/s10529-012-1012-6, PMID: 22850791

[ref23] Beara I. N. Lesjak M. M. Jovin E. Đ. Balog K. J. Anackov G. T. Orcic D. Z.. (2009). Plantain (*Plantago* L.) species as novel sources of flavonoid antioxidants. J. Agric. Food Chem. 57, 9268–9273. doi: 10.1021/jf902205m, PMID: 19754195

[ref24] Beara I. N. Lesjak M. M. Orčić D. Z. Simin N. Đ. Četojević-Simin D. D. Božin B. N.. (2012). Comparative analysis of phenolic profile, antioxidant, anti-inflammatory and cytotoxic activity of two closely-related plantain species: *Plantago altissima* L. and *Plantago lanceolata* L. LWT-Food Sci. Technol. 47, 64–70. doi: 10.1016/j.lwt.2012.01.001

[ref25] Behbahani B. A. Shahidi F. Yazdi F. T. Mohebbi M. (2013). Antifungal effect of aqueous and ethanolic mangrove plant extract on pathogenic fungus “in vitro”. Int. J. Agron. Plant Prod. 4, 1652–1658. doi: 10.17795/zjrms-5992

[ref26] Bhardwaj S. K. (2012). Evaluation of plant extracts as antifungal agents against *Fusarium solani* (Mart.) Sacc. World J. Agric. Sci. 8, 385–388.

[ref27] Biradar S. R. Rachetti B. D. (2013). Extraction of some secondary metabolites & thin layer chromatography from different parts of *Centella asiatica* L. (URB). Am. J. Life Sci. 1, 243–247. doi: 10.11648/j.ajls.20130106.11

[ref28] Chandrasekaran M. Chun S. C. (2016). Expression of PR-protein genes and induction of defense-related enzymes by *Bacillus subtilis* CBR05 in tomato (*Solanum lycopersicum*) plants challenged with *Erwinia carotovora* subsp. carotovora. Biosci. Biotechnol. Biochem. 80, 2277–2283. doi: 10.1080/09168451.2016.1206811, PMID: 27405462

[ref29] Channa M. Y. Pathan M. A. Solangi G. R. Wondiar M. (1995). Studies on *Rhizoctonia solani* (Kuhn) causing root rot of lentil. Sarhad J. Agric 11, 495–499.

[ref30] Chiang L. C. Chiang W. Chang M. Y. Ng L. T. Lin C. C. (2002). Antiviral activity of *Plantago major* extracts and related compounds in vitro. Antivir. Res. 55, 53–62. doi: 10.1016/S0166-3542(02)00007-4, PMID: 12076751

[ref31] Çoban T. Çitoǧlu G. S. Sever B. İşcan M. (2003). Antioxidant activities of plants used in traditional medicine in Turkey. Pharm. Biol. 41, 608–613. doi: 10.1080/13880200390501974

[ref32] Culver M. Fanuel T. Chiteka A. Z. (2012). Effect of moringa extract on growth and yield of tomato. Greener J. Agric. Sci. 2, 207–211. doi: 10.5281/zenodo.3372890

[ref33] D’Maris Amick Dempsey A. C. Vlot M. C. W. Daniel F. K. Dempsey D. A. Vlot A. C. Wildermuth M. C.. (2011). Salicylic acid biosynthesis and metabolism. Arab. Book 9:e0156. doi: 10.1199/tab.0156 PMC3268552 22303280

[ref34] Deena M. J. Thoppil J. E. (2000). Antimicrobial activity of the essential oil of *Lantana camara*. Fitoterapia 71, 453–455. doi: 10.1016/S0367-326X(00)00140-4, PMID: 10925025

[ref35] Desoky E.-S. M. ElSayed A. I. Merwad A.-R. M. A. Rady M. M. (2019). Stimulating antioxidant defenses, antioxidant gene expression, and salt tolerance in *Pisum sativum* seedling by pretreatment using licorice root extract (LRE) as an organic biostimulant. Plant Physiol. Biochem. 142, 292–302. doi: 10.1016/j.plaphy.2019.07.020, PMID: 31351320

[ref36] Dhingra O. D. Sinclair J. B. (2017). Basic Plant Pathology Methods. Boca Raton, FL: CRC Press.

[ref37] Dissanayake M. (2014). Inhibitory effect of selected medicinal plant extracts on phytopathogenic fungus *Fusarium oxysporum* (Nectriaceae) Schlecht. Emend. Snyder and Hansen. Annu. Res. Rev. Biol. 4, 133–142. doi: 10.9734/ARRB/2014/5777

[ref38] Druzhinina I. S. Seidl-Seiboth V. Herrera-Estrella A. Horwitz B. A. Kenerley C. M. Monte E.. (2011). Trichoderma: the genomics of opportunistic success. Nat. Rev. Microbiol. 9, 749–759. doi: 10.1038/nrmicro2637, PMID: 21921934

[ref39] El-Benawy N. M. Abdel-Fattah G. M. Ghoneem K. M. Shabana Y. M. (2020). Antimicrobial activities of *Trichoderma atroviride* against common bean seed-borne *Macrophomina phaseolina* and *Rhizoctonia solani*. Egypt. J. Basic Appl. Sci. 7, 267–280. doi: 10.1080/2314808X.2020.1809849

[ref40] El-Kazzaz M. K. Ghoneim K. E. Agha M. K. M. Helmy A. Behiry S. I. Abdelkhalek A.. (2022). Suppression of pepper root rot and wilt diseases caused by *Rhizoctonia solani* and *Fusarium oxysporum*. Life 12, 587. doi: 10.3390/life12040587, PMID: 35455078 PMC9029026

[ref41] Ertani A. Pizzeghello D. Francioso O. Tinti A. Nardi S. (2016). Biological activity of vegetal extracts containing phenols on plant metabolism. Molecules 21, 205. doi: 10.3390/molecules21020205, PMID: 26867189 PMC6273273

[ref42] Ferreira C. Oliveira R. (2020). Protective antifungal activity of *Plantago major* extract against the phytopathogenic fungi *Phytophthora cinnamomi*, *Diplodia corticola* and *Colletotrichum* species. *Proceedings*, 70:94. doi: 10.3390/foods_2020-07678

[ref43] Gálvez M. Martín-Cordero C. Houghton P. J. Ayuso M. J. (2005). Antioxidant activity of methanol extracts obtained from *Plantago* species. J. Agric. Food Chem. 53, 1927–1933. doi: 10.1021/jf048076s 15769115

[ref44] Gondal A. S. Rauf A. Naz F. (2019). Anastomosis groups of *Rhizoctonia solani* associated with tomato foot rot in Pothohar Region of Pakistan. Sci. Rep. 9, 1–12. doi: 10.1038/s41598-019-40043-5 30846707 PMC6405938

[ref45] Hamza A. Mohamed A. Derbalah A. (2016). Unconventional alternatives for control of tomato root rot caused by *Rhizoctonia solani* under greenhouse conditions. J. plant Prot. Res. 56, 298–305. doi: 10.1515/jppr-2016-0046

[ref46] Heflish A. A. Abdelkhalek A. Al-Askar A. A. Behiry S. I. (2021). Protective and curative effects of *Trichoderma asperelloides* Ta41 on tomato root rot caused by *Rhizoctonia solani* Rs33. Agronomy 11, 1162. doi: 10.3390/agronomy11061162

[ref47] Hoegen E. Strömberg A. Pihlgren U. Kombrink E. (2002). Primary structure and tissue-specific expression of the pathogenesis-related protein PR-1b in potato. Mol. Plant Pathol. 3, 329–345. doi: 10.1046/j.1364-3703.2002.00126.x, PMID: 20569341

[ref48] Hof H. (2001). Critical annotations to the use of azole antifungals for plant protection. Antimicrob. Agents Chemother. 45, 2987–2990. doi: 10.1128/AAC.45.11.2987-2990.2001, PMID: 11600346 PMC90772

[ref49] James J. Dubery I. (2011). Identification and quantification of triterpenoid centelloids in *Centella asiatica* (L.) urban by densitometric TLC. J. Planar Chromatogr. 24, 82–87. doi: 10.1556/JPC.24.2011.1.16

[ref50] Jat J. G. Agalave H. R. (2013). Fungitoxic properties of some leaf extracts against oilseed-borne fungi. Sci. Res. Report 3, 210–215.

[ref51] Karima S. Farida S. Mihoub Z. M. (2015). Antioxidant and antimicrobial activities of *Plantago major*. Int. J. Pharm. Pharm. Sci. 7, 58–64.

[ref52] Klironomos J. N. (2003). Variation in plant response to native and exotic arbuscular mycorrhizal fungi. Ecology 84, 2292–2301. doi: 10.1890/02-0413

[ref53] Koka J. A. Wani A. H. Bhat M. Y. Parveen S. (2017). Antagonistic activity of *Trichoderma* spp. against some fungi causing fungal rot disease of brinjal. Trends Biosci. 10, 2844–2846.

[ref54] Kumar A. Shukla R. Singh P. Prasad C. S. Dubey N. K. (2008). Assessment of *Thymus vulgaris* L. essential oil as a safe botanical preservative against post harvest fungal infestation of food commodities. Innov. Food Sci. Emerg. Technol. 9, 575–580. doi: 10.1016/j.ifset.2007.12.005

[ref55] Kuprashvili T. D. (1996). The use of phytoncides for seed treatment. Zas. Karantin Rast. 5:31.

[ref56] Livak K. J. Schmittgen T. D. (2001). Analysis of relative gene expression data using real- time quantitative PCR and the 2 Ϫ ⌬⌬ C T method. Methods 25, 402–408. doi: 10.1006/meth.2001.1262 11846609

[ref57] Lucas G. B. Campbell C. L. Lucas L. T. (1992). Introduction to Plant Diseases: Identification and Management. New York, NY: Springer Science & Business Media

[ref58] Mani J. S. Johnson J. B. Steel J. C. Broszczak D. A. Neilsen P. M. Walsh K. B.. (2020). Natural product-derived phytochemicals as potential agents against coronaviruses: a review. Virus Res. 284:197989. doi: 10.1016/j.virusres.2020.197989, PMID: 32360300 PMC7190535

[ref59] Marais J. P. J. Deavours B. Dixon R. A. Ferreira D. (2006). “The stereochemistry of flavonoids,” in The science of flavonoids, Grotewold E. (Berlin: Springer Press, 1–46.

[ref60] Martin N. Vesentini D. Rego C. Monteiro S. Oliveira H. Ferreira R. B. (2009). *Phaeomoniella chlamydospora* infection induces changes in phenolic compounds content in *Vitis vinifera*. Phytopathol. Mediterr. 48, 101–116. doi: 10.14601/Phytopathol_Mediterr-2879

[ref61] Martínez G. Regente M. Jacobi S. Del Rio M. Pinedo M. de la Canal L. (2017). Chlorogenic acid is a fungicide active against phytopathogenic fungi. Pestic. Biochem. Physiol. 140, 30–35. doi: 10.1016/j.pestbp.2017.05.012, PMID: 28755691

[ref62] Mikulic-Petkovsek M. Schmitzer V. Stampar F. Veberic R. Koron D. (2014). Changes in phenolic content induced by infection with *Didymella applanata* and *Leptosphaeria coniothyrium*, the causal agents of raspberry spur and cane blight. Plant Pathol. 63, 185–192. doi: 10.1111/ppa.12081

[ref63] Miser-Salihoglu E. Akaydin G. Caliskan-Can E. Yardim-Akaydin S. (2013). Evalution of antioxidant activity of various herbal folk medicines. J. Nutr. Food Sci, 3–222. doi: 10.4172/2155-9600.1000222

[ref64] Mohamed A. A. Behiry S. I. Ali H. M. EL-Hefny M. Salem M. Z. M. Ashmawy N. A. (2020). Phytochemical compounds of branches from *P. halepensis* oily liquid extract and *S. terebinthifolius* essential oil and their potential antifungal activity. Processes 8, 330. doi: 10.3390/pr8030330

[ref65] Montealegre J. Valderrama L. Sánchez S. Herrera R. Besoain X. Pérez L. M. (2010). Biological control of *Rhizoctonia solani* in tomatoes with *Trichoderma harzianum* mutants. Electron. J. Biotechnol. 13, 1–2. doi: 10.2225/vol13-issue2-fulltext-6

[ref66] Moorthy K. K. Subramaniam P. Senguttuvan J. (2013). In vitro antifungal activity of various extracts of leaf and stem parts of *Solena amplexicaulis* (Lam.) Gandhi. Int. J. Pharm. Pharm. Sci. 5, 745–747.

[ref67] Mvumi C. Tagwira F. Chiteka A. Z. (2013). Effect of moringa extract on growth and yield of maize and common beans. Greener J. Agric. Sci. 3, 55–62. doi: 10.15580/GJAS.2013.1.111512264

[ref68] Nazzaro F. Fratianni F. Coppola R. De Feo V. (2017). Essential oils and antifungal activity. Pharmaceuticals 10, 86. doi: 10.3390/ph10040086, PMID: 29099084 PMC5748643

[ref69] Nishibe S. Murai M. Tamayama Y. (1995). Studies on constituents of Plantaginis Herba 7: flavonoids from *Plantago asiatica* and *P. hostifolia*. Nat. Med. 49, 340–342.

[ref70] Niu D.-D. Liu H.-X. Jiang C.-H. Wang Y.-P. Wang Q.-Y. Jin H.-L.. (2011). The plant growth–promoting rhizobacterium *Bacillus cereus* AR156 induces systemic resistance in *Arabidopsis thaliana* by simultaneously activating salicylate-and jasmonate/ethylene-dependent signaling pathways. Mol. Plant-Microbe Interact. 24, 533–542. doi: 10.1094/MPMI-09-10-0213, PMID: 21198361

[ref71] Parveen S. Wani A. H. Bhat M. Y. Malik A. R. Koka J. A. Ashraf N. (2017). Antimycotic potential of some phytoextracts on some pathogenic fungi. J. Biopest. 10, 60–65.

[ref72] Parveen S. Wani A. H. Ganie A. A. Pala S. A. Mir R. A. (2014). Antifungal activity of some plant extracts on some pathogenic fungi. Arch. Phytopathol. Plant Prot. 47, 279–284. doi: 10.1080/03235408.2013.808857

[ref73] Petkovsek M. M. Slatnar A. Stampar F. Veberic R. (2011). Phenolic compounds in apple leaves after infection with apple scab. Biol. Plant. 55, 725–730. doi: 10.1007/s10535-011-0176-6

[ref74] Prasannath K. (2017). Plant defense-related enzymes against pathogens: a review. AGRIEAST J. Agric. Sci. 11, 38. doi: 10.4038/agrieast.v11i1.33

[ref75] Rex J. H. Pfaller M. A. Rinaldi M. G. Polak A. Galgiani J. N. (1993). Antifungal susceptibility testing. Clin. Microbiol. Rev. 6, 367–381. doi: 10.1128/CMR.6.4.367, PMID: 8269392 PMC358294

[ref76] Rex J. H. Rinaldi M. G. Pfaller M. A. (1995). Resistance of *Candida* species to fluconazole. Antimicrob. Agents Chemother. 39, 1–8. doi: 10.1128/AAC.39.1.1, PMID: 7695288 PMC162475

[ref77] Roylawar P. Panda S. Kamble A. (2015). Comparative analysis of BABA and *Piriformospora indica* mediated priming of defence-related genes in tomato against early blight. Physiol. Mol. Plant Pathol. 91, 88–95. doi: 10.1016/j.pmpp.2015.06.004

[ref78] Rusjan D. Veberič R. Mikulič-Petkovšek M. (2012). The response of phenolic compounds in grapes of the variety ‘Chardonnay’ (*Vitis vinifera* L.) to the infection by phytoplasma Bois noir. Eur. J. Plant Pathol. 133, 965–974. doi: 10.1007/s10658-012-9967-7

[ref79] Sabuquillo P. De Cal A. Melgarejo P. (2006). Biocontrol of tomato wilt by *Penicillium oxalicum* formulations in different crop conditions. Biol. Control 37, 256–265. doi: 10.1016/j.biocontrol.2006.02.009

[ref80] Salem M. Z. M. Behiry S. I. EL-Hefny M. (2019). Inhibition of *Fusarium culmorum*, *Penicillium chrysogenum* and *Rhizoctonia solani* by n-hexane extracts of three plant species as a wood-treated oil fungicide. J. Appl. Microbiol. 126, 1683–1699. doi: 10.1111/jam.14256, PMID: 30887609

[ref81] Samuelsen A. B. (2000). The traditional uses, chemical constituents and biological activities of *Plantago major* L. A review. J. Ethnopharmacol. 71, 1–21. doi: 10.1016/S0378-8741(00)00212-9, PMID: 10904143 PMC7142308

[ref82] Santas J. Almajano M. P. Carbó R. (2010). Antimicrobial and antioxidant activity of crude onion (*Allium cepa*, L.) extracts. Int. J. Food Sci. Technol. 45, 403–409. doi: 10.1111/j.1365-2621.2009.02169.x

[ref83] Schneiter R. Di Pietro A. (2013). The CAP protein superfamily: function in sterol export and fungal virulence. Biomol. Concepts 4, 519–525. doi: 10.1515/bmc-2013-0021, PMID: 25436594

[ref84] Shaygannia E. Bahmani M. Zamanzad B. Rafieian-Kopaei M. (2016). A review study on *Punica granatum* L. J. Evid. Based. Complement. Altern. Med. 21, 221–227. doi: 10.1177/2156587215598039 26232244

[ref85] Shimada K. Fujikawa K. Yahara K. Nakamura T. (1992). Antioxidative properties of xanthan on the autoxidation of soybean oil in cyclodextrin emulsion. J. Agric. Food Chem. 40, 945–948. doi: 10.1021/jf00018a005

[ref86] Shuping D. S. S. Eloff J. N. (2017). The use of plants to protect plants and food against fungal pathogens: a review. African J. Tradit. Complement. Altern. Med. 14, 120–127. doi: 10.21010/ajtcam.v14i4.14, PMID: 28638874 PMC5471458

[ref87] Silva P. A. Oliveira D. F. do Prado N. R. T. de Carvalho D. A. de Carvalho G. A. (2008). Evaluation of the antifungal activity by plant extracts against *Colletotrichum gloeosporioides* PENZ. Ciênc. Agrotecnol. 32, 420–428. doi: 10.1590/S1413-70542008000200012

[ref88] Singh D. Singh P. Gupta A. Solanki S. Sharma E. Nema R. (2012). Qualitative estimation of the presence of bioactive compound in *Centella asiatica*: an important medicinal plant. Int. J. Life Sci. Med. Sci. 2, 4–7.

[ref89] Singh R. P. Singh P. K. Rutkoski J. Hodson D. P. He X. Jørgensen L. N.. (2016). Disease impact on wheat yield potential and prospects of genetic control. Annu. Rev. Phytopathol. 54, 303–322. doi: 10.1146/annurev-phyto-080615-095835, PMID: 27296137

[ref90] Singh B. N. Singh A. Singh S. P. Singh H. B. (2011). *Trichoderma harzianum*-mediated reprogramming of oxidative stress response in root apoplast of sunflower enhances defence against *Rhizoctonia solani*. Eur. J. Plant Pathol. 131, 121–134. doi: 10.1007/s10658-011-9792-4

[ref91] Su Z.-Z. Mao L.-J. Li N. Feng X.-X. Yuan Z.-L. Wang L.-W.. (2013). Evidence for biotrophic lifestyle and biocontrol potential of dark septate endophyte *Harpophora oryzae* to rice blast disease. PLoS One 8:e61332. doi: 10.1371/journal.pone.0061332, PMID: 23637814 PMC3630206

[ref92] Valarini P. J. de Melo I. S. Morsoletto R. V. (2003). Alternative control of root rot of common beans (*Phaseolus vulgaris* L.). Summa Phytopathol. 29, 334–339.

[ref93] Velázquez-Fiz M. P. Díaz-Lanza A. M. Fernández-Matellano L. (2000). Iridoids from *Plantago lagopus*. Pharm. Biol. 38, 268–270. doi: 10.1076/1388-0209(200009)3841-AFT268, PMID: 21214473

[ref94] Velioglu Y. Mazza G. Gao L. Oomah B. D. (1998). Antioxidant activity and total phenolics in selected fruits, vegetables, and grain products. J. Agric. Food Chem. 46, 4113–4117. doi: 10.1021/jf9801973

[ref95] Wu Z. Huang Y. Li Y. Dong J. Liu X. Li C. (2019). Biocontrol of *Rhizoctonia solani* via induction of the defense mechanism and antimicrobial compounds produced by *Bacillus subtilis* SL-44 on pepper (*Capsicum annuum* L.). Front. Microbiol. 10:2676. doi: 10.3389/fmicb.2019.02676, PMID: 31849858 PMC6892779

